# Positive aspect of caregiving among primary informal dementia caregivers in Singapore

**DOI:** 10.1371/journal.pone.0237677

**Published:** 2020-08-20

**Authors:** Fiona Devi, Qi Yuan, Peizhi Wang, Gregory Tee Hng Tan, Richard Roshan Goveas, Li Ling Ng, Siow Ann Chong, Mythily Subramaniam

**Affiliations:** 1 Research Division, Institute of Mental Health, Singapore, Singapore; 2 Department of Geriatric Psychiatry, Institute of Mental Health, Singapore, Singapore; 3 Department of Psychological Medicine, Changi General Hospital, Singapore, Singapore; Texas Technical University Health Sciences Center, UNITED STATES

## Abstract

**Background:**

The present study investigated the factor structure of positive aspects of caregiving (PAC) scale among primary informal caregivers providing care to persons with dementia (PWD) in Singapore.

**Methods:**

282 primary informal caregivers of PWD were recruited from the Institute of Mental Health, and Changi General Hospital and administered the 9-item PAC scale. A confirmatory factor analyses (CFA) was conducted to test the model fit of the 9-item PAC proposed by the scale developer and multiple linear regression was used to investigate the significant socio-demographic correlates.

**Results:**

CFA showed that the 2-factor structure including ‘Self-Affirmation’ and ‘Outlook on Life’ had an acceptable model fit. After controlling for confounding variables, Malay caregivers were associated with higher scores on PAC and ‘Self-Affirmation’ compared to caregivers of other ethnicities. Caregivers with Secondary or below education level had higher PAC and ‘Outlook on Life’ scores. Caregivers who had received formal training scored higher in PAC, ‘Self-Affirmation’ and ‘Outlook on Life’.

**Discussion:**

The present study confirmed that the 2-factor structure of the 9-item PAC was suitable for informal caregivers of PWD in Singapore. The findings have important implications for locally available interventions to enhance caregiver’s psychological well-being and reduce burden of care.

## Introduction

The Diagnostic and Statistical Manual of Mental Disorders 5^th^ Edition (DSM-5), defines dementia as major Neurocognitive Disorder (NCD) which is characterised by an acquired, progressive deterioration of cognitive functions [1–3] that leads to impairment in the person’s activities of daily living (e.g., bathing, talking, eating, shopping, paying the bills) [4] and neuropsychiatric symptoms such as agitation, depression and hallucinations [4]. Hence, majority of persons with dementia (PWD) are significantly dependent on the informal caregivers such as their family, relatives or friends for their care [5, 6].

According to the World Alzheimer's report in 2010, the cost of informal caregiving (e.g., unpaid care provided by families, informal caregivers and others) contributed almost 40 to 60 percent worldwide (US$604 billion) of the total cost of dementia [7, 8]. In Singapore, the total cost of dementia was estimated to be SG$532 million [9]. The mean annual cost of informal caregiving for PWD in Singapore was 1.7 times higher as compared to formal caregiving (SG$44,530.55 versus SG$25,654.11), which was largely contributed from the time informal caregivers spent with the PWD [10]. In addition, caregivers are often required to cope with the illness; where many are unfamiliar and are even unprepared to recognise the symptoms and adapt to the changes in behaviour of the PWD [11]. The role of caregiving involves bringing PWD for treatments, making decisions on their behalf; overseeing medications, paying for the expenses, liaising with various healthcare professionals, coping with PWD’s behavioural problems etc., thus playing a very important role in the care plan for PWD [12]. Over a period of time, such responsibilities may result in financial burden and caregivers neglecting their mental and physical wellbeing thus reducing their quality of life [13, 14]. However, many of them remain filial and appreciate the positive sides of their role as they consider it their responsibility and duty to care for the PWD [15].

Thus, despite all the challenges, caregiving has been positively associated with a perceived sense of satisfaction, improved personal resilience, self- efficacy and a sense of purpose in life [12, 16, 17]. The positive aspect of caregiving (PAC) was defined as "the gains or satisfaction resulting from the caregiving experience" [17]. Studies have reported that in spite of the stress, almost 80% of caregivers experienced positive caregiving (e.g., personal growth, sharing activities, strengthened relationship with PWD, increase in self-esteem, and spirituality) [6, 17–20].

Lawton et al. [21] proposed a 2-factor model of caregiving appraisal and psychological well-being in caregiving experience whereby positive caregiving experiences that are perceived as rewarding serve as a coping mechanism against negative effects. Besides improving caregivers' life satisfaction and quality of life, these components also influence their involvement in providing a better quality of care to the PWD [22, 23].

Within the caregiving experience, positive aspects seem to be influenced by several factors. David et al. [24] showed that socio-demographic differences among caregivers such as age, race, gender, education level, health and relationship status of the informal caregiver with the PWD play a role in the caregiving experiences. Caregivers are often women, usually spouse, who generally provide more hours of care and have reported higher physical burden, financial strain, depression symptoms and lower psychological wellbeing as compared to adult-child caregivers [25]. Specifically, wives and female adult-child caregivers have reported less positive experiences as compared to husband and male adult-child caregivers [26]. Baker and Robertson [27] reported that older age group male caregivers and lower family income caregivers had a more positive caregiving experience.

One widely used scale for measuring psychological benefits of caregiving among caregivers is the PAC scale [28]. A 11-item PAC scale was used in the initial REACH I project by Tarlow and colleagues, which was later found to be psychometrically valid through exploratory factor analysis (EFA), and confirmatory factory analysis (CFA). As such, they proposed the 9-item PAC scale, consisting of 2-factors (Self Affirmation; six items); (Outlook on Life; three items) and an overall PAC score. Both the 9-item and 11-item PAC scale have been used extensively in many countries [29–32]. A study by Siow et al. [31] assessed the validity and reliability of the 9- item PAC scale among a representative sample of caregivers of older Singaporeans adults with functional limitations. The 9-item PAC scale showed acceptable content and constructs high validity as well as cross-language reliability indices, similarly to other studies [12, 33].

Many studies [12, 17, 29, 32] have examined the relationship between PAC and its influence on caregivers well-being or role in providing care. However, the majority of these studies involved Western populations. Hence, there is a need for local studies to examine and recognize factors associated with PAC especially in a multi-ethnic country such as Singapore which can help to identify and provide insight into planning interventions for caregivers' well-being. Thus, in view of these findings our study aims to (1) investigate the applicability of the 2-factor structure of the 9-item PAC scale among informal caregivers of PWD in Singapore; and (2) explore the different factors associated with PAC among primary informal caregivers providing care to PWD in Singapore.

## Methods

### Participants and procedures

A total of 433 informal caregivers of PWD were approached from the Institute of Mental Health (IMH) as well as its satellite clinics, and Changi General Hospital (CGH) Singapore from January 2017 to December 2018. A total of 282 informal caregivers agreed to participate representing a response rate of 65%. Inclusion criteria of the study included: 1) Singapore citizens or Permanent Resident aged 21 years and above; 2) being the primary informal caregiver to a patient who has been formally diagnosed with dementia; 3) able to speak English, Chinese, or Malay language. Exclusion criteria included those who had difficulty understanding the informed consent and those who did not visit the PWD minimally at least once on a weekly basis.

Participants volunteered by responding to posters in the outpatient clinics or were referred by clinicians who were study team members. Sociodemographic information and a series of questionnaires were administered by study team members through a face to face interview. Upon completion of the questionnaires, participants were reimbursed with SGD 40 cash as an inconvenience fee.

The ethical approval for the study was approved by the National Healthcare Group Domain Specific Review Board in Singapore (reference number: 2016/00921). Written informed consent was obtained from all participants prior to their enrolment and appropriate measures were taken to ensure confidentiality and data privacy.

### Translation and back translation

The official language used in Singapore is English, however majority of the older generation are more fluent communicating in their native language such as Chinese or Malay. Hence, the 9-item PAC scale was translated into both Chinese and Malay language using a standard ‘translation back-translation’ technique [34]. The translation process started with translating the English items into written Chinese or Malay language by a bilingual research assistant, followed by back -translating the languages into the English items by another bilingual research assistant. Quality of the translations were checked by the principal investigator and co- investigators by assessing the similarity of the original set and the back-translated set of the English items as well as the grammatical and conceptual accuracy of the language items.

### Instruments

#### Sociodemographic questionnaire

Socio-demographic information comprising age, gender, ethnicity, marital status, educational level and hours of caregiving were collected using a structured questionnaire.

#### The Positive Aspects of Caregiving (PAC)

The PAC is a 9-item validated instrument designed to measure the perceived positive aspects of caregiving [28]. Informal caregivers were asked to indicate their agreement with 9 statements about possible positive experiences associated with the caregiving. Each item began with the sentence "Providing help to [care recipient] has…," followed with specific items such as "made me feel useful" and "enabled me to develop a more positive attitude toward life." The subscale was measured by rating on a 5-point Likert scale, ranging from 1 (disagree a lot) to 5 (agree a lot). A higher score indicates a more positive caregiving experience. There were no reverse-scored items.

### Statistical analyses

CFA was conducted to test the model fit of the 9-item PAC to ensure validity among the informal caregivers of dementia patient in Singapore through the “lavaan” package under R software [35]. In the present study, a good model is described as 1) the comparative fit index (CFI) > 0.95; and 2) the Tucker-Lewis index (TLI) > 0.95; and 3) the root mean square error of approximation (RMSEA) < 0.08 [36]. In addition, internal reliability was evaluated using Cronbach’s alpha. Descriptive statistics were tabulated for the socio-demographics of the sample and other dependent variables. After controlling for confounding variables, multiple linear regression model was used to investigate the factors associated with PAC among the informal caregivers. Sociodemographic characteristics and socioeconomic status of informal caregivers, relationship with PWD, PWD’s current living status, availability of domestic helper and attending formal training on caregiving were examined. Descriptive statistics and multiple linear regression were tabulated using the SAS 9.3. Statistical significance was reported at P < .05 (2-tailed) throughout the study.

## Results

Sociodemographic characteristics of the participants are shown in Table 1. A total of 282 participants comprising a majority of female caregivers (75.2%), of Chinese ethnicity (83%), ever married (72%), and with Secondary and below education (42.5%) constituted the sample. In terms of socioeconomic status, majority were employed (57.1%) and had a monthly income ranging from SGD 2,000 to SGD 5,999 (27.66%). Participants were mainly daughter caregivers (55.32%), currently living with the care recipient (70.21%) and did not have a domestic helper hired specifically for the care recipient (56.4%). In addition, most of the participants (75.9%) had not received any formal training on caregiving. Prior to further analysis, CFA was conducted to test if the factor structure proposed by the scale developer [28] fit our caregiver sample. Results suggested that the 2-factor structure showed an acceptable model fit–CFI = 0.992, TLI = 0.989, RMSEA = 0.091 (above 0.08 but still shows mediocre fit) [37]. The first factor consisting of 6 items was labelled as ‘Self-Affirmation’ with statements pertaining to affirmation of oneself in dementia caregiving. The second factor consisting 3 items was labelled as ‘Outlook on Life’. The mean PAC factors scores among the informal caregivers for ‘Self-Affirmation’ was 23.34 (SD = 4.78), and for ‘Outlook on Life’ was 12.03 (SD = 2.56) (Table 2). The average PAC was 35.37 (SD = 6.58). The internal reliability statistics (Cronbach’s alpha) for the PAC, ‘Self-Affirmation’ and ‘Outlook on Life’ were 0.883, 0.873, and 0.785 respectively.

**Table 1:** Socio-demographic characteristics.

Variables		Informal Caregivers (n = 282)
Age, mean ± SD		55.70 ± 11.84
Gender, N (%)		
	Male	70 (24.82)
	Female	212 (75.18)
Ethnicity, N (%)		
	Chinese	234 (83.0)
	Malay	29 (10.28)
	Indian & Others	19 (6.74)
Marital status, N (%)		
	Never married	79 (28.01)
	Ever married	203 (72.0)
Education level, N (%)		
	Secondary or below	120 (42.6)
	‘A’ Level/Diploma/ Other diploma	73 (25.9)
	Degree or above	89 (31.6)
Employment status, N (%)		
	Unemployed/ retired/ housewife	121 (42.9)
	Employed	161 (57.1)
Personal monthly income, N (%)		
	<SGD 2,000	56 (19.9)
	SGD 2,000- SGD5, 999	78 (27.7)
	SGD 6,000 or above	42 (14.9)
	Not applicable	106 (37.6)
Relationship with PWD		
	Spouse	43 (15.3)
	Son	48 (17.0)
	Daughter	156 (55.3)
	Others	35 (12.4)
Current living status with PWD		
	Yes	198 (70.2)
	No	84 (29.8)
Domestic Helper hired specifically for PWD		
	Yes	159 (56.4)
	No	123 (43.6)
Formal training in Caregiving		
	Yes	68 (24.1)
	No	214 (75.9)

Continuous variables were listed as mean and standard deviation (SD); for categorical variables, they were presented as frequency and percentage.

The Singapore-Cambridge General Certificate of Education Advanced Level (A level); Singapore dollars (SGD); Persons with Dementia (PWD).

**Table 2:** PAC factor scores among informal caregivers with PWD.

	Informal Caregivers			
	Mean	SD	Score Range	
			**Min**	**Max**
**PAC**	35.37	6.58	9	45
**Self-Affirmation**	23.34	4.78	6	30
**Outlook on Life**	12.03	2.56	3	15

Positive Aspect of Caregiving (PAC); Persons with Dementia (PWD).

The present study also examined the correlates of the overall PAC and those of the two factors (see Table 3). Multiple linear regression results were tabulated after adjusting for confounding variables. The results showed that Malay informal caregivers (versus Chinese) were found to be significantly and positively associated with higher PAC (β = 2.94, 95% CI [0.20 to 5.68]) and ‘Self-Affirmation’ (β = 2.37, 95% CI [0.38 to 4.36]) scores. On the other hand, informal caregivers with Secondary or below education level (versus university and above) had higher PAC (β = 2.41, 95% CI [0.31 to 4.51]) and ‘Outlook on Life’ (β = 0.95, 95% CI [0.13 to 1.76]) scores. Similarly, informal caregivers with ‘A’ Level or Diploma education level (versus university and above) had higher PAC (β = 1.04, 95 CI [0.20 to 1.88]) scores. Those who had attended formal training in caregiving (versus no formal training) were associated with significantly higher PAC (β = 2.57, 95% CI [0.65 to 4.49]), ‘Self-Affirmation’ (β = 1.48, 95% CI [0.08 to 2.87]) and ‘Outlook on Life’ (β = 1.09, 95% CI [0.35 to 1.83]) scores. No significant associations were found between the PAC factors and informal caregivers’ relationship with PWD.

**Table 3:** Factors associated with PAC and informal caregivers sociodemographic variables.

	PAC				Self- Affirmation				Outlook on Life			
Sociodemographic characteristics	β	95% CI		p-value	β	95% CI		p-value	β	95% CI		p-value
**Age**	0.02	-0.07	0.11	0.72	0.01	-0.05	0.08	0.69	0.00	-0.03	0.04	0.85
**Gender**												
Male	1.9	-1.54	5.34	0.28	1.63	-0.87	4.13	0.19	0.27	-1.06	1.6	0.69
Female	Ref				Ref				Ref			
**Ethnicity**												
Indian & Others	1.37	-1.88	4.61	0.41	0.81	-1.55	3.17	0.49	0.56	-0.69	1.82	0.38
Malay	2.94	0.20	5.68	0.04	2.37	0.38	4.36	0.02	0.57	-0.49	1.63	0.29
Chinese	Ref				Ref				Ref			
**Marital status**												
Never married	-1.08	-2.99	0.82	0.26	-0.99	-2.38	0.39	0.16	-0.09	-0.83	0.65	0.81
Ever married	Ref				Ref				Ref			
**Education level**												
Secondary or below	2.41	0.31	4.51	0.03	1.46	-0.07	2.99	0.06	0.95	0.13	1.76	0.02
‘A’ Level/Diploma/ Other diploma	2.15	-0.00	4.31	0.05	1.12	-0.45	2.68	0.16	1.04	0.20	1.88	0.02
Degree or above	Ref				Ref				Ref			
**Employment status**												
Unemployed/ retired/ housewife	-0.79	-3.73	2.15	0.59	-0.24	-2.38	1.89	0.82	-0.55	-1.68	0.59	0.34
Employed	Ref				Ref				Ref			
**Personal monthly income**												
Not applicable	1.98	-1.24	5.19	0.23	0.88	-1.46	3.21	0.46	1.10	-0.14	2.34	0.08
SGD 2,000- SGD5, 999	1.28	-1.12	3.68	0.29	0.48	-1.27	2.22	0.59	0.8	-0.13	1.73	0.09
SGD 6,000 or above	1.51	-1.45	4.48	0.32	0.44	-1.72	2.59	0.69	1.08	-0.07	2.23	0.07
<SGD 2,000	Ref				Ref				Ref			
**Relationship with PWD**												
Others	1.05	-2.64	4.74	0.58	-0.04	-2.72	2.64	0.98	1.09	-0.34	2.51	0.14
Son	0.55	-3.39	4.49	0.78	-0.28	-3.14	2.59	0.85	0.83	-0.69	2.35	0.29
Daughter	1.11	-2.2	4.42	0.51	0.38	-2.02	2.79	0.75	0.72	-0.56	2.00	0.27
Spouse	Ref				Ref				Ref			
**Current living status with PWD**												
Yes	0.29	-1.55	2.14	0.75	0.23	-1.10	1.57	0.73	0.06	-0.65	0.77	0.87
No	Ref				Ref				Ref			
**Domestic Helper hired specifically for PWD**												
Yes	-0.28	-1.91	1.35	0.74	-0.22	-1.41	0.96	0.71	-0.06	-0.69	0.58	0.86
No	Ref				Ref				Ref			
**Formal training in Caregiving**												
Yes	2.57	0.65	4.49	0.01	1.48	0.08	2.87	0.04	1.09	0.35	1.83	0.00
No	Ref				Ref				Ref			

β (Beta coefficient) was derived using multiple linear regression analyses after adjusting for all covariates (sociodemographic characteristics and socioeconomic status of informal caregivers, relationship with PWD, PWD’s current living status, availability of domestic helper and attending formal training on caregiving).

The Singapore-Cambridge General Certificate of Education Advanced Level (A level); Singapore dollars (SGD); Positive Aspect of Caregiving (PAC), Persons with Dementia (PWD).

## Discussion

The present study aimed to explore the different factors associated with PAC among primary informal caregivers providing care to PWD in Singapore. Similar to the study by Tarlow et al. [28], the present study confirmed that the 2-factor structure comprising ‘Self-Affirmation’ and ‘Outlook on Life’ of the 9-item PAC was applicable to informal caregivers of PWD in Singapore.

Several studies [38–40] have reported the disadvantages that caregivers with lower education face such as inability to regulate negative emotions or stress, formulate coping strategies, finding available resources and getting easily depressed. However, the present study found that informal caregivers with lower education reported higher PAC and better ‘Outlook on Life’ in their caregiving roles. Similarly, other studies [41, 42] have suggested that caregivers with lower education are positively associated with a lower caregiving burden due to a more simplistic mind-set or lifestyle as compared to those with a higher academic multitasking lifestyle. In addition, with a simple daily life, the need for caregivers to sacrifice their daily habits is less likely which unfortunately often occurs in dementia caregiving [41].

Cultural differences often influence the relationships between caregivers’ caregiving experiences and their roles. Current findings showed that Malay informal caregivers were more likely to have a positive perception of caregiving experiences as compared to informal caregivers belonging to other ethnicities. One possible explanation could be related to cultural and religious beliefs that are more strongly valued among Malay informal caregivers. In Singapore, majority of the Malay population are predominantly Muslim, hence established Islamic ethics and practices are dominant in this group [43, 44]. Historically in the Islamic teaching, suffering, illness and challenges are perceived as trials from God by which one’s sins are removed if endured with patience and resilience [45]. In addition, caring for older adults, not just parents is also considered a religious and moral obligation in the Muslim community [46].

On the basis of previous research [47–50], the present study had anticipated that immediate family caregivers (e.g., adult- child or spouse) would report lower PAC as compared to distant family caregivers (e.g., siblings, nephews, niece, etc.). Reasons for such differences include immediate family caregivers experiencing a higher level of emotional responsibility (e.g. sense of guilt and social pressure), psychological distress and stronger tradition (e.g., filial piety) among collectivist societies [51, 52]. However, no significant associations were found between PAC and the informal caregivers’ relationship status with PWD in the present study.

Current findings also show that informal caregivers who attended formal training in caregiving reported high levels of PAC and both factors (‘Self-Affirmation’ and ‘Outlook on Life’). In the early stages, families or caregivers of PWD are often unprepared to handle the challenges such as decision making, managing the level of care, communicating effectively that commonly follows after the person has been a diagnosed to have dementia [53]. Caregivers lack of understanding, poor access to information and available support causes knowledge gap which affects and delays both caregiver and PWD’s adjustment to the diagnosis [11, 53]. This result in caregivers having a harder time in making sense of the changes the PWD is experiencing [11]. Hence, training programs, interventions and information can contribute to a better caregiving experience, quality of life and mental wellbeing for caregivers and even decrease the risk of early institutionalization for the PWD [54]. The findings of the present study support the notion that formal training for informal caregivers may play a crucial role in PAC.

### Limitations

There are some limitations in this study. Firstly, the study was limited to informal caregivers of patients who attended outpatient clinics, hence the findings cannot be generalised to all informal caregivers of PWD in the community. Secondly, clinical status of PWD (e.g., stages or severity of dementia, behavioural problems) was not collected, which could affect caregiver’s burden. Thus, we were not able to determine if PAC is dependent on the different stages of caregiving or on the severity of dementia that informal caregivers may experience throughout their caregiving journey. Lastly, as the questionnaire was interviewer- administered, informal caregivers might have been influenced by social desirability bias in an effort to be viewed favourably.

## Conclusion

In conclusion, the present study confirmed that the PAC scale had the same 2-factor structure among informal caregivers of PWD in Singapore as proposed by Tarlow et al. [28]. Results demonstrated that the 9-item PAC scale comprises two distinct components of caregiving experience: Self-Affirmation and Outlook on Life. Overall, our findings underscore the need for training and educational interventions in improving PAC levels for informal caregivers. An earlier study in Singapore established that a pilot mobile application based psycho-educational intervention, resulted in significant reduction in perceived burden, and distress while positive emotional outcomes improved among caregivers of PWDs [55]. Hence, it is possible that adopting more positive caregiving strategies and attending caregiver training or educational interventions can improve psychological well-being among caregivers.

## References

[1] Sachdev PS, Mohan A, Taylor L, Jeste DV. DSM-5 and Mental Disorders in Older Individuals: An Overview. Harv Rev Psychiatry. 2015;23(5):320–8. 10.1097/HRP.0000000000000090 26332215 PMC4562208

[2] Gauthier S, Reisberg B, Zaudig M, Petersen RC, Ritchie K, Broich K, et al Mild cognitive impairment. Lancet (London, England). 2006;367(9518):1262–70. 10.1016/S0140-6736(06)68542-5 16631882

[3] Association AP. Diagnostic and statistical manual of mental disorders Arlington (VA): American Psychiatric Publishing; 2013.

[4] Duong S, Patel T, Chang F. Dementia: What pharmacists need to know. Can Pharm J (Ott). 2017;150(2):118–29. 28405256 10.1177/1715163517690745 PMC5384525

[5] Zwaanswijk M, Peeters JM, van Beek AP, Meerveld JH, Francke AL. Informal caregivers of people with dementia: problems, needs and support in the initial stage and in subsequent stages of dementia: a questionnaire survey. Open Nurs J. 2013;7:6–13. 10.2174/1874434601307010006 23346266 PMC3551235

[6] Fauziana R, Sambasivam R, Vaingankar JA, Abdin E, Ong HL, Tan M-E, et al Positive Caregiving Characteristics as a Mediator of Caregiving Burden and Satisfaction With Life in Caregivers of Older Adults. J Geriatr Psychiatry Neurol. 2018;31(6):329–35. 10.1177/0891988718802111 30260715 PMC6262596

[7] Prince M, Bryce R, Albanese E, Wimo A, Ribeiro W, Ferri CP. The global prevalence of dementia: A systematic review and metaanalysis. Alzheimer's & Dementia: The Journal of the Alzheimer's Association. 2013;9(1):63–75.e2. 10.1016/j.jalz.2012.11.007 23305823

[8] Prince M., Wimo A., Guerchet M., Gemma-Claire Ali, Yu-Tzu Wu, Prina M. The Global Impact of Dementia: An analysis of prevalence, incidence, cost and trends Alzheimer ‘s Disease International 2015.

[9] Abdin E, Subramaniam M, Achilla E, Chong SA, Vaingankar JA, Picco L, et al The Societal Cost of Dementia in Singapore: Results from the WiSE Study. Journal of Alzheimer's disease: JAD. 2016;51(2):439–49. 10.3233/JAD-150930 26890766

[10] Woo LL, Thompson CL, Magadi H. Monetary cost of family caregiving for people with dementia in Singapore. Archives of gerontology and geriatrics. 2017;71:59–65. 10.1016/j.archger.2017.03.006 28347930

[11] Robinson L, Clare L, Evans K. Making sense of dementia and adjusting to loss: psychological reactions to a diagnosis of dementia in couples. Aging & mental health. 2005;9(4):337–47. 16019290 10.1080/13607860500114555

[12] Grover S, Nehra R, Malhotra R, Kate N. Positive Aspects of Caregiving Experience among Caregivers of Patients with Dementia. East Asian archives of psychiatry: official journal of the Hong Kong College of Psychiatrists = Dong Ya jing shen ke xue zhi: Xianggang jing shen ke yi xue yuan qi kan. 2017;27(2):71–8. 28652500

[13] Sullivan AB, Miller D. Who is Taking Care of the Caregiver? J Patient Exp. 2015;2(1):7–12. 10.1177/237437431500200103 28725810 PMC5513610

[14] Schulz R, Czaja SJ. Family Caregiving: A Vision for the Future. The American journal of geriatric psychiatry: official journal of the American Association for Geriatric Psychiatry. 2018;26(3):358–63. 28774786 10.1016/j.jagp.2017.06.023

[15] Wong O, Chau B. The Evolving Role of Filial Piety in Eldercare in Hong Kong 2006 600–17 p.

[16] Cohen CA, Gold DP, Shulman KI, Zucchero CA. Positive Aspects in Caregiving: An Overlooked Variable in Research. Canadian Journal on Aging / La Revue canadienne du vieillissement. 1994;13(3):378–91.

[17] Cohen CA, Colantonio A, Vernich L. Positive aspects of caregiving: rounding out the caregiver experience. International journal of geriatric psychiatry. 2002;17(2):184–8. 10.1002/gps.561 11813283

[18] Boerner K, Schulz R, Horowitz A. Positive aspects of caregiving and adaptation to bereavement. Psychology and aging. 2004;19(4):668–75. 10.1037/0882-7974.19.4.668 15584791

[19] Center NOR. Long term care in America: Expectations and realities. 2014. Available from: http://www.longtermcarepoll.org/PDFs/LTC%202014/AP-NORC-Long-Term%20Care%20in%20America_FINAL%20WEB.pdf.

[20] Sanders S. Is the Glass Half Empty or Half Full? Social Work in Health Care. 2005;40(3):57–73. 10.1300/J010v40n03_04 15837668

[21] Lawton MP, Moss M, Kleban MH, Glicksman A, Rovine M. A two-factor model of caregiving appraisal and psychological well-being. Journal of gerontology. 1991;46(4):P181–9. 10.1093/geronj/46.4.p181 2071844

[22] Carbonneau H, Caron C, Desrosiers J. Development of a conceptual framework of positive aspects of caregiving in dementia. Dementia. 2010;9(3):327–53.

[23] Roth DL, Perkins M, Wadley VG, Temple EM, Haley WE. Family caregiving and emotional strain: associations with quality of life in a large national sample of middle-aged and older adults. Quality of life research: an international journal of quality of life aspects of treatment, care and rehabilitation. 2009;18(6):679–88. 10.1007/s11136-009-9482-2 PMC2855243 19421895

[24] David L. R, Fredman L, Haley WE. Informal Caregiving and Its Impact on Health: A Reappraisal From Population-Based Studies. The Gerontologist. 2015;55(2):309–19. 10.1093/geront/gnu177 26035608 PMC6584119

[25] Pinquart M, Sorensen S. Spouses, adult children, and children-in-law as caregivers of older adults: a meta-analytic comparison. Psychology and aging. 2011;26(1):1–14. 10.1037/a0021863 21417538 PMC4449135

[26] Lin IF, Fee HR, Wu H-S. Negative and Positive Caregiving Expriences: A Closer Look at the Intersection of Gender and Relationships. Fam Relat. 2012;61(2):343–58. 10.1111/j.1741-3729.2011.00692.x 22544989 PMC3335398

[27] Baker KL, Robertson N. Coping with caring for someone with dementia: reviewing the literature about men. Aging & mental health. 2008;12(4):413–22. 18791888 10.1080/13607860802224250

[28] Tarlow BJ, Wisniewski SR, Belle SH, Rubert M, Ory MG, Gallagher-Thompson D. Positive Aspects of Caregiving: Contributions of the REACH Project to the Development of New Measures for Alzheimer’s Caregiving. Research on Aging. 2004;26(4):429–53.

[29] Cho J, Ory MG, Stevens AB. Socioecological factors and positive aspects of caregiving: findings from the REACH II intervention. Aging & mental health. 2016;20(11):1190–201. 26213337 10.1080/13607863.2015.1068739

[30] Lou VW, Lau BH, Cheung KS. Positive aspects of caregiving (PAC): scale validation among Chinese dementia caregivers (CG). Archives of gerontology and geriatrics. 2015;60(2):299–306. 10.1016/j.archger.2014.10.019 25488014

[31] Siow JYM, Chan A, Østbye T, Cheng GH-L, Malhotra R. Validity and Reliability of the Positive Aspects of Caregiving (PAC) Scale and Development of Its Shorter Version (S-PAC) Among Family Caregivers of Older Adults. The Gerontologist. 2017;57(4):e75–e84. 10.1093/geront/gnw198 28082275

[32] Kate N, Grover S, Kulhara P, Nehra R. Scale for positive aspects of caregiving experience: development, reliability, and factor structure. East Asian archives of psychiatry: official journal of the Hong Kong College of Psychiatrists = Dong Ya jing shen ke xue zhi: Xianggang jing shen ke yi xue yuan qi kan. 2012;22(2):62–9. 22714876

[33] Abdollahpour I, Nedjat S, Salimi Y. Positive Aspects of Caregiving and Caregiver Burden: A Study of Caregivers of Patients With Dementia. J Geriatr Psychiatry Neurol. 2018;31(1):34–8. 10.1177/0891988717743590 29187025

[34] Jones PS, Lee JW, Phillips LR, Zhang XE, Jaceldo KB. An adaptation of Brislin's translation model for cross-cultural research. Nursing research. 2001;50(5):300–4. 10.1097/00006199-200109000-00008 11570715

[35] Rosseel Y. lavaan: An R Package for Structural Equation Modeling. 2012. 2012;48(2):36.

[36] Lt Hu, Bentler PM. Cutoff criteria for fit indexes in covariance structure analysis: Conventional criteria versus new alternatives. Structural Equation Modeling: A Multidisciplinary Journal. 1999;6(1):1–55.

[37] MacCallum RC, Browne MW, Sugawara HM. Power analysis and determination of sample size for covariance structure modeling. Psychological Methods. 1996;1(2):130–49.

[38] Tzeng N-S, Chang C-W, Hsu J-Y, Chou Y-C, Chang H-A, Kao Y-C. Caregiver Burden for Patients with Dementia with or Without Hiring Foreign Health Aides: A Cross-Sectional Study in a Northern Taiwan Memory Clinic. Journal of Medical Sciences. 2015;35(6):239–47.

[39] Olazarán Rodríguez J, Sastre Paz M, Martín Sánchez S. Health care in dementia: Satisfaction and needs of the caregiver. Neurología (English Edition). 2012;27(4):189–96. 10.1016/j.nrl.2011.07.005 21903303

[40] Omranifard V, Haghighizadeh E, Akouchekian S. Depression in Main Caregivers of Dementia Patients: Prevalence and Predictors. Advanced Biomedical Research. 2018;7(1):34–. 29531932 10.4103/2277-9175.225924 PMC5841004

[41] Raivio M, Laakkonen M, Strandberg T, Savikko N, Tilvis R, Eloniemi-Sulkava U, et al Gender Differences in Dementia Spousal Caregiving 2012 162960 p. 10.1155/2012/162960 PMC3465980 23056990

[42] Rapp SR, Chao D. Appraisals of strain and of gain: Effects on psychological wellbeing of caregivers of dementia patients. Aging & mental health. 2000;4(2):142–7.

[43] Alghafli Z, Hatch T, Marks L. Religion and Relationships in Muslim Families: A Qualitative Examination of Devout Married Muslim Couples. Religions. 2014;5(3):814–33.

[44] Mathew M, Khidzer, M.K.B. and Key, T.K. Religiosity and the Management of Religious Harmony: Responses from the IPS Survey on Race, Religion and Language. 2014. Contract No.: IPS Working Papers No 2129

[45] Choong KA. Islam and palliative care. Global Bioethics. 2015;26(1):28–42.

[46] Kristine J, Ajrouch. Caring for Aging Muslim Families: A Needs Assessment. Institute for Social Policy and Understanding; 2016.

[47] Kim D. Relationships between Caregiving Stress, Depression, and Self-Esteem in Family Caregivers of Adults with a Disability. Occup Ther Int. 2017;2017:1686143–. 10.1155/2017/1686143 29114184 PMC5664279

[48] Norton MC, Smith KR, Østbye T, Tschanz JT, Corcoran C, Schwartz S, et al Greater risk of dementia when spouse has dementia? The Cache County study. J Am Geriatr Soc. 2010;58(5):895–900. 10.1111/j.1532-5415.2010.02806.x 20722820 PMC2945313

[49] Amirkhanyan AA, Wolf DA. Caregiver Stress and Noncaregiver Stress: Exploring the Pathways of Psychiatric Morbidity. The Gerontologist. 2003;43(6):817–27. 10.1093/geront/43.6.817 14704381

[50] Brodaty H, Donkin M. Family caregivers of people with dementia. Dialogues Clin Neurosci. 2009;11(2):217–28. 19585957 10.31887/DCNS.2009.11.2/hbrodaty PMC3181916

[51] Paulson D, Lichtenberg PA. Effect of Caregiver Family Status on Care Recipient Symptom Severity and Caregiver Stress at Nursing Home Intake. Clinical gerontologist. 2011;34(2):132–43. 10.1080/07317115.2011.539518 21796229 PMC3142942

[52] Lai DW. Cultural predictors of caregiving burden of Chinese-Canadian family caregivers. Canadian journal on aging = La revue canadienne du vieillissement. 2007;26 Suppl 1:133–47. 18089531 10.3138/cja.26.suppl_1.133

[53] Whitlatch CJ, Orsulic-Jeras S. Meeting the Informational, Educational, and Psychosocial Support Needs of Persons Living With Dementia and Their Family Caregivers. The Gerontologist. 2018;58(suppl_1):S58–S73. 10.1093/geront/gnx162 29361068

[54] Smits CH, de Lange J, Droes RM, Meiland F, Vernooij-Dassen M, Pot AM. Effects of combined intervention programmes for people with dementia living at home and their caregivers: a systematic review. International journal of geriatric psychiatry. 2007;22(12):1181–93. 10.1002/gps.1805 17457793

[55] Chunxiang X. A Psycho- Educational Intervention for Family Caregivers of Patients with Dementia using A Mobile Application: A Pilot Randomized Controlled Trial: National University of Singapore; 2016.

